# Enteric Duplication Cysts as a Diagnostic and Therapeutic Challenge: Three Unique Case Reports With Review of Literature

**DOI:** 10.7759/cureus.99418

**Published:** 2025-12-16

**Authors:** Yaramala Sai Bharath Reddy, Pradeep K Jena, Varsha Madhavnarayan Totadri, Subrat K Mohanty, Harish Chandra Tudu, Sruti Mohanty, Amaresh Mishra

**Affiliations:** 1 General Surgery, Kalinga Institute of Medical Sciences, Bhubaneswar, IND; 2 Paediatric Surgery, Kalinga Institute of Medical Sciences, Bhubaneswar, IND

**Keywords:** alimentary tract, common wall, diagnostic dilemma, enteric duplication cyst, paediatric

## Abstract

Enteric duplication cysts (EDCs) are rare congenital anomalies that may occur anywhere along the gastrointestinal tract. They are typically located adjacent to the alimentary canal, sharing a common muscular wall and sometimes a lumen. The conundrum arises in the diagnosis due to difficulty in identifying the anatomical abnormality via simple radiological modalities, thereby delaying intervention. Their clinical presentation varies with location and complications. While enteric duplication cysts are uncommon, they can present with non-specific and potentially life-threatening symptoms in infancy. Accurate preoperative diagnosis requires high clinical suspicion and advanced imaging modalities, as routine investigations may appear normal. Early recognition and surgical management of EDCs are essential to prevent complications. Antenatal detection allows for early planning and surgical management. In this series, we report three pediatric cases of enteric duplication cysts presenting with distinct complications. The first case was a seven-day-old female neonate with bilious vomiting, abdominal distension, and feed intolerance since birth. Despite a normal abdominal radiograph, contrast-enhanced CT revealed a terminal ileal duplication cyst compressing the ileocecal junction and causing obstruction. The second case was a six-month-old male infant, with an undocumented antenatal suspicion of enteric cyst, presenting with acute abdomen and radiological features of perforation peritonitis. Laparotomy revealed a perforated, isolated jejunal duplication cyst. The third case was a seven-month-old female infant with recurrent intussusception, where hydrostatic reduction identified a terminal ileal duplication cyst serving as the lead point. All patients underwent successful surgical excision, with histopathological confirmation of EDC, and recovered uneventfully. This case series highlights the varied presentation, diagnostic dilemma, and timely management required for successful outcomes of enteric duplication cysts.

## Introduction

Enteric duplication cysts are rare, congenital, epithelial-lined cystic structures with an incidence of 1:4500 live births [1]. These developmental anomalies can arise in variable locations along the gastrointestinal tract, sharing a common blood supply and intestinal wall [2]. Most enteric duplication cysts do not communicate with the lumen of the adjacent bowel. 20-30% of enteric duplication cysts contain heterotopic tissue, such as gastric mucosa or pancreatic tissue, that contributes to the clinical presentation [3-6].

With the advent of stringent antenatal evaluation and the expedited use of prenatal anomaly scans, enteric duplication cysts are commonly detected in utero, thereby necessitating specific management in the postnatal period [7-10]. In those undiagnosed antenatally, they pose a dilemma both diagnostically and therapeutically due to the non-specific and varied modalities of presentation [9,10]. The clinical manifestations depend upon the site, size, and type of cyst [11,12]. Common clinical presentations include abdominal distension, intolerance to feeds, bilious vomiting, and gastrointestinal bleeding, with complicated cases presenting with intussusception and perforation peritonitis [11-14].

With the ease and availability of imaging modalities such as contrast-enhanced computed tomography (CECT), the diagnostic process has been streamlined, further aiding in careful decision-making for surgical intervention [15-18]. The confounding factor is primarily the nonspecific and occasionally delayed presentation, masking the pathology and exposing patients to months of medical management [19]. With a better understanding of the propensity to identify this anomaly primarily in the neonatal period and infancy, surgical management can be tailored [18,19].

Each case in this series of three cases is presented in a different age group with varied symptoms-the first one presenting with features of neonatal intestinal obstruction, the second with perforation peritonitis, and the third with recurrent intussusception (caused by a lead point). The arborizing clinical presentations in each form the baseline of this series, with the aim of highlighting challenges faced in the diagnosis and management of EDCs.

## Case presentation

Case 1

**Figure 1:**
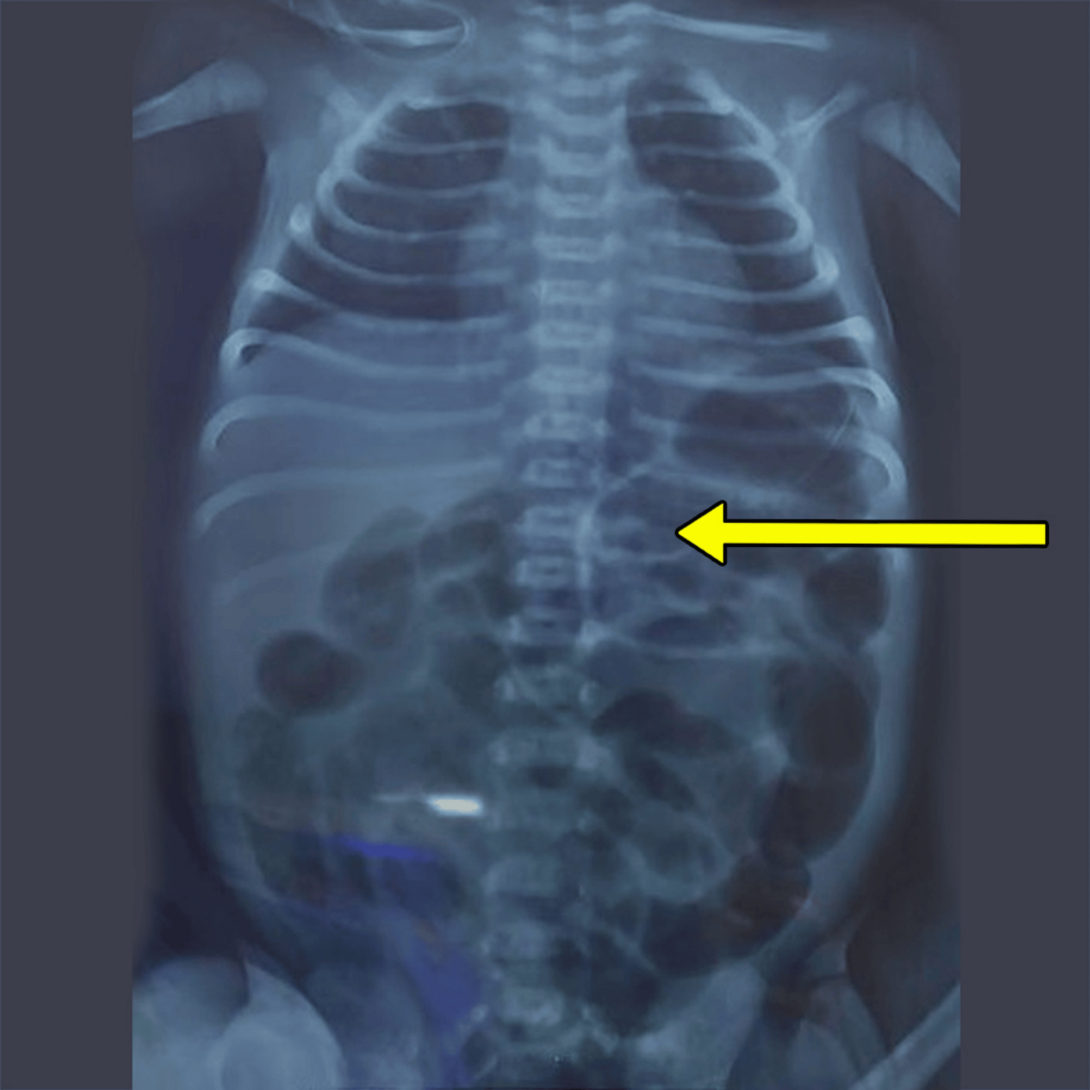
X-ray of the abdomen and pelvis showing a normal bowel gas pattern (yellow arrow)

A seven-day-old female neonate with no significant antenatal findings presented with poor feeding, bilious vomiting, and abdominal distension for one day prior. With an initial suspicion of midgut volvulus, the patient was admitted to the neonatal intensive care unit (NICU), resuscitated, and evaluated. An abdominal radiograph revealed a normal gas shadow pattern (Figure 1).

**Figure 2:**
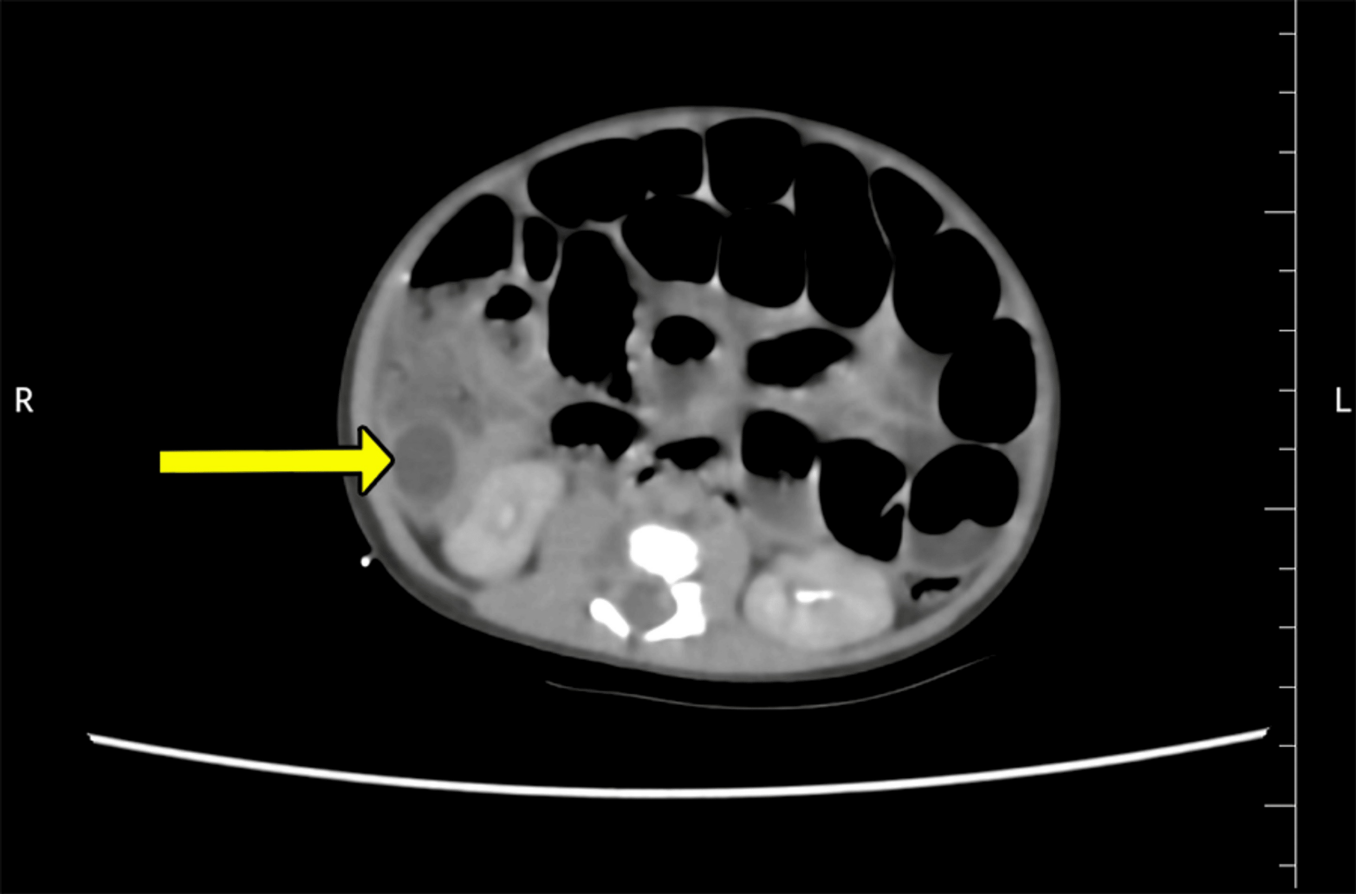
Contrast-enhanced computed tomography (CECT) of the abdomen and pelvis Demonstrating a well-defined cystic lesion in the terminal ileum, abutting the cecum and causing obstruction (yellow arrow)

Ultrasonography (USG) of the abdomen and pelvis showed an intramural cystic lesion of 1.6x1.4x1.6 cm with internal debris, closely associated with a loop of distal ileum, causing severe luminal narrowing. A provisional diagnosis of enteric duplication cyst with intestinal obstruction was made, and for better delineation of anatomy, a contrast-enhanced computed tomography of the abdomen and pelvis (CECT) was done, which showed a well-defined cystic lesion of size 1.5x1.1x1.8cm abutting the cecum and terminal ileal loops, bulging into the cecum, with intestinal obstruction (Figure 2). 

**Figure 3:**
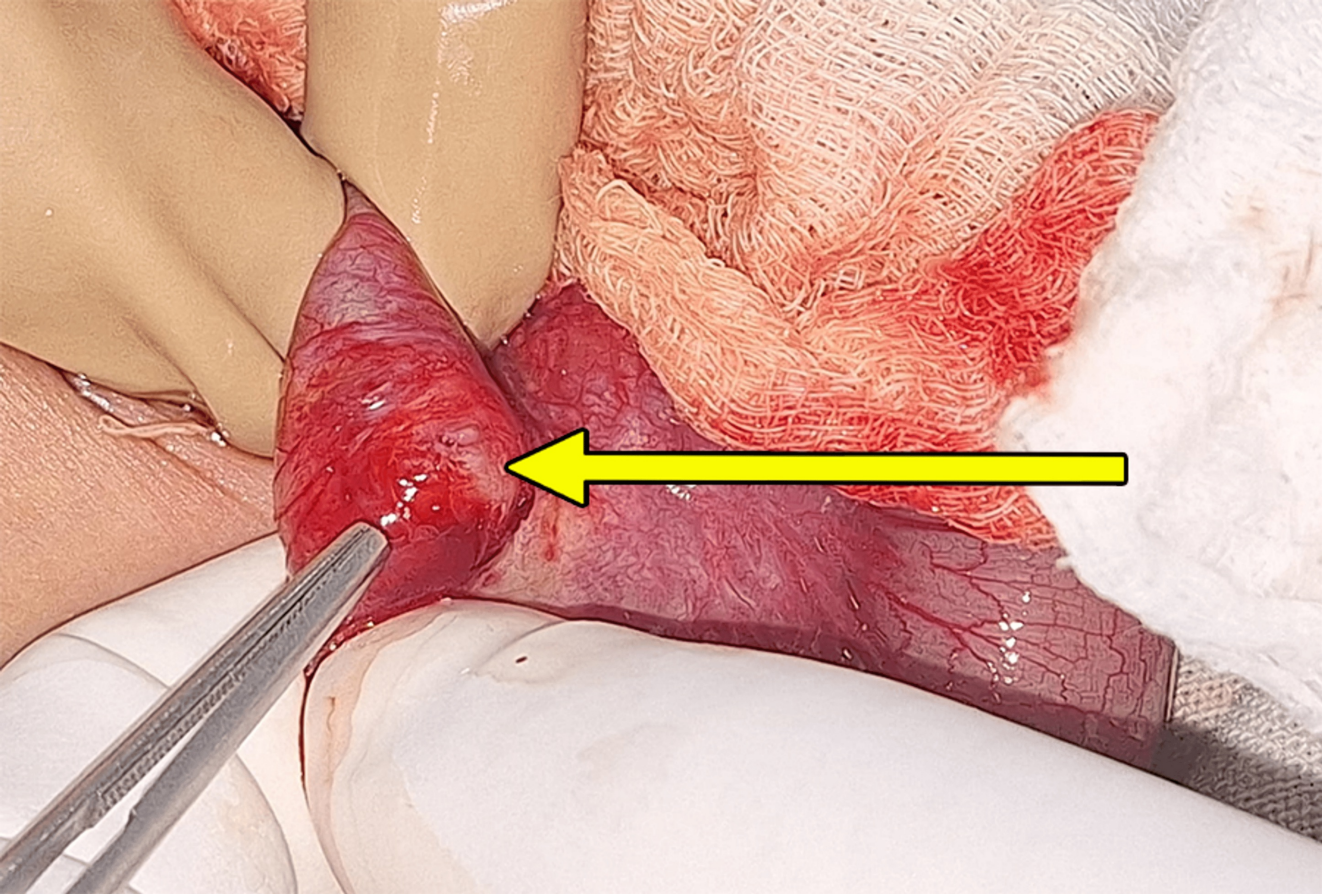
Intraoperative finding of Enteric duplication cyst (EDC) arising from terminal ileum (yellow arrow)

The child was taken up for an exploratory laparotomy wherein intraoperatively a 2x2 cm enteric duplication cyst was identified arising from the terminal ileum, 3.5 cm proximal to the ileocecal junction (ICJ), sharing a common wall and lumen with the associated bowel (Figure 3).

Excision of the cyst along with the adjacent ileum and end-to-end anastomosis was done, preserving the ICJ. The baby had an uneventful postoperative period and was gradually started on oral feeds from postoperative day (POD) 4 and was discharged on POD 10. The histopathology report was suggestive of a cyst lined by intestinal epithelium with a shared muscular wall with the terminal ileum, with no other ectopic mucosa. The baby is thriving well and continues to remain on regular outpatient follow-up.

Case 2

**Figure 4:**
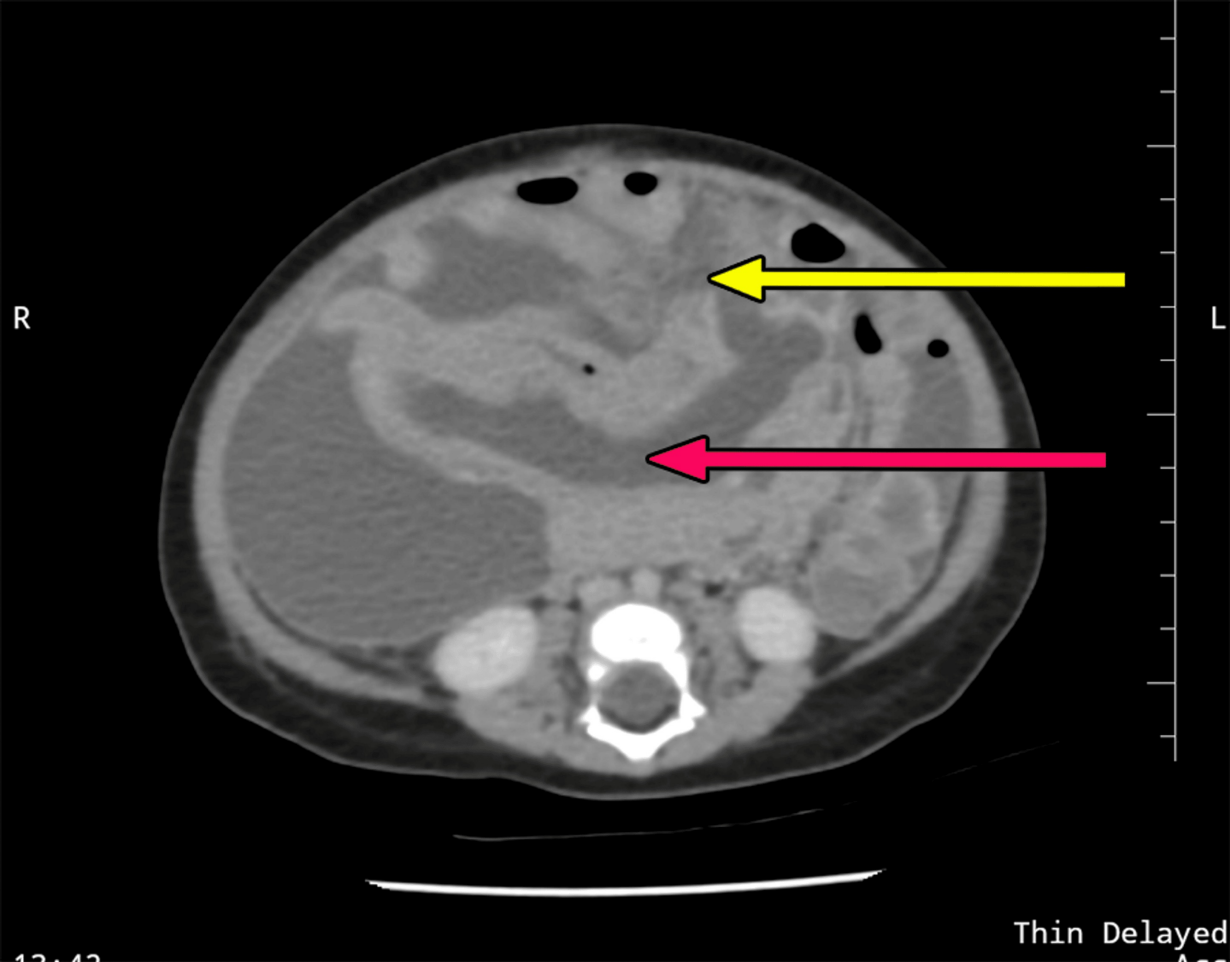
Contrast-enhanced computed tomography (CECT) of the abdomen and pelvis Demonstrating a thick-walled jejunal duplication cyst (red arrow) with a defect in its anterior wall (yellow arrow), suggestive of perforation

A six-month-old male infant presented to the emergency room with progressive abdominal distension, bilious vomiting, and constipation for one day. The child was antenatally diagnosed with a suspicious intestinal cyst at 28 weeks period of gestation, with no documented antenatal reports available. No postnatal evaluation was done. On examination, the abdomen was distended with diffuse tenderness and guarding. The child was admitted to the pediatric intensive care unit (PICU) for resuscitation. An abdominal radiograph showed grossly dilated bowel loops, and an abdominal and pelvic ultrasound revealed a suspicious cystic lesion adjacent to a small bowel loop with surrounding air pockets with gross intraperitoneal collection with internal septations-likely perforation. With the given clinical picture and radiological findings, a strong likelihood of a complicated enteric duplication cyst was considered, and on stabilization, an emergency CECT of the abdomen and pelvis was done, which showed a thick-walled cystic lesion within the jejunal mesentery with a defect in its anterior wall, showing communication with intraperitoneal collection-likely a ruptured enteric duplication cyst with peritonitis (Figure 4).

**Figure 5:**
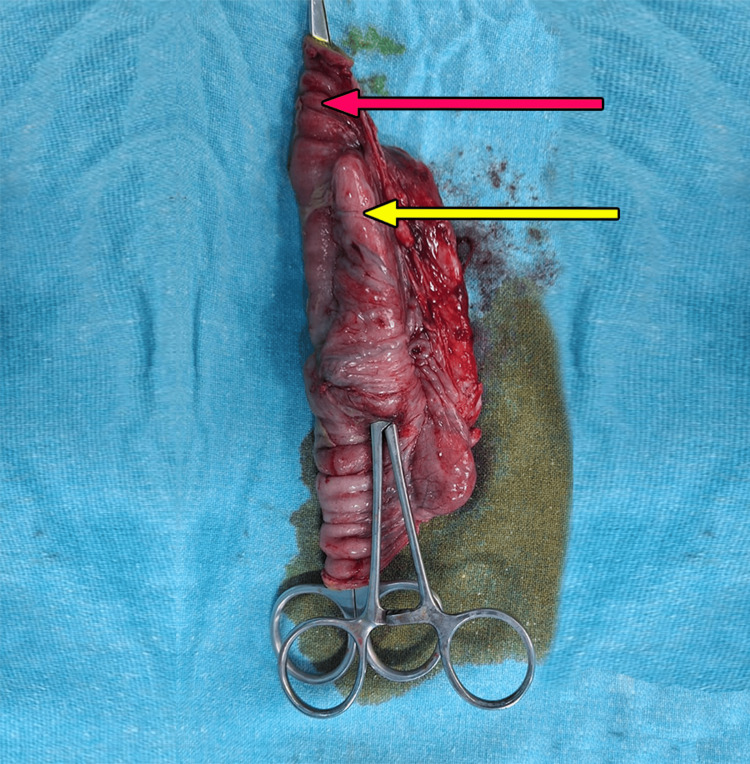
Intraoperative excised specimen of bowel with enteric duplication cyst Demonstrating a normal jejunal bowel loop (red arrow) with an adjacent enteric duplication cyst (yellow arrow), sharing a common wall

The child underwent an emergency exploratory laparotomy. Intraoperatively, there was biliary peritonitis with an enteric duplication cyst of size 5x2 cm, identified in the jejunum, 25 cm distal to the duodenojejunal flexure (DJF), along the mesenteric side, with perforation over its anterior wall (Figure 5).

Resection of 10 cm of afflicted bowel along with the EDC was done with end-to-end anastomosis and thorough peritoneal lavage. The child was managed in the PICU postoperatively with supportive fluids and antibiotics to phase out the sepsis. Nasogastric aspirate gradually reduced, and the child was started on oral feeds from POD 6. Histopathological analysis showed a cyst wall containing gastric epithelium and intestinal mucosa with a common shared wall between the associated small bowel and cyst, with extensive exudation from the cyst. The child gradually improved and was discharged on POD 12 and continues to remain on follow-up on an outpatient basis.

Case 3

**Figure 6:**
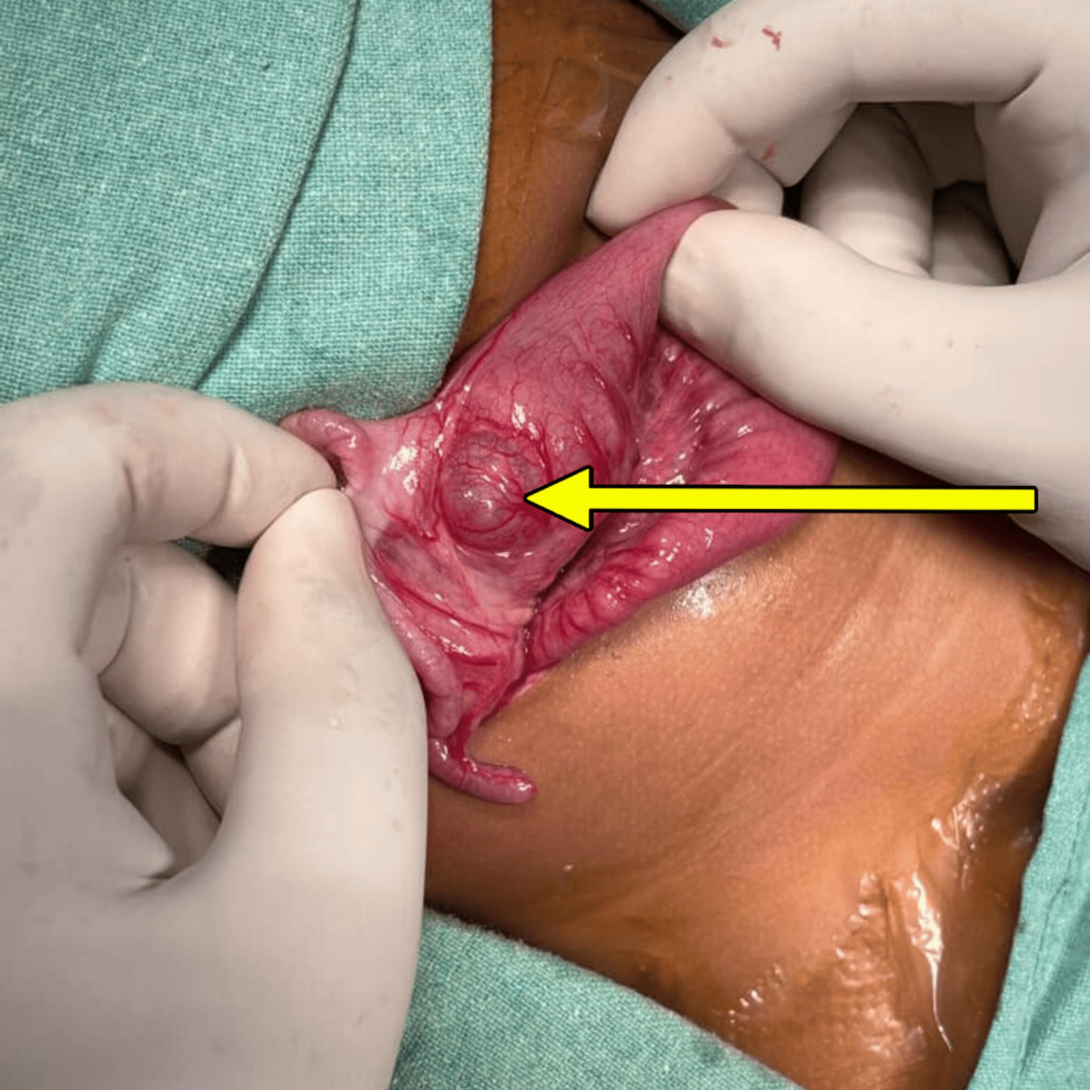
Intraoperative finding of enteric duplication cyst just proximal to ileocecal junction (yellow arrow)

A seven-month-old female infant presented to the emergency department with complaints of abdominal pain and bleeding per rectum for two days. On examination, the abdomen was soft with no tenderness, but a per rectal examination revealed red currant jelly stools. With a provisional diagnosis of intussusception, resuscitation and radiological investigations were done, which confirmed ileocolic intussusception of 3.5 cm in length. The patient underwent USG-guided hydrostatic reduction of the intussusception, which was successful. One day after the procedure, the child again developed severe abdominal pain, with a repeat ultrasonography showing recurrent intussusception. A second sitting of hydrostatic reduction was done, which was successful, but thereafter a finding of a cystic lesion of 3x2 cm was identified in the terminal ileum in continuity with the bowel loop, strongly suggestive of an ileal duplication cyst, possibly acting as a lead point for the intussusception. The child underwent an exploratory laparotomy, where an ileal duplication cyst of 3x2 cm was identified just proximal to the ileocecal junction (Figure 6).

Excision of the cyst with limited resection of the bowel was done (along with ICJ), and ileo-ascending colon anastomosis was done. The child was shifted to the PICU and placed on continuous NG aspiration. Oral intake was initiated on postoperative day (POD) 5 and progressively increased. The child thereafter had an excellent recovery. The histopathology report confirmed a cyst wall containing intestinal mucosa and having a common shared wall with the adjacent bowel loop, with no other ectopic mucosa.

## Discussion

Enteric duplication cysts (EDC) are extremely rare, congenital anomalies that can present with potentially life-threatening complications if not diagnosed appropriately [1]. They are identified in only 0.2% of children [1,2]. Due to its varied location along the entire length of the alimentary canal, the clinical presentations may range from subtle nonspecific symptoms to critical surgical emergencies [2,3]. Most duplication cysts are detected in children within the first year of life, with antenatal detection taking precedence in recent practice [4,5]. While the exact etiology for the development of enteric duplication cysts is not definitively known, there remain multiple postulations, with the most commonly accepted being the split notochord theory, with others including partial/abortive twinning, persistent embryological diverticula, and aberrant luminal recanalization [5-7].

EDCs are structurally classified into cystic or tubular forms, with the cystic variety being more common [1,2,5]. Cystic EDC does not communicate with the adjacent lumen, while the tubular form communicates with the associated gastrointestinal tract [6-8]. With respect to the location of EDC, the most common site was identified to be the ileum in various literature (60%), followed by colonic, jejunal, gastric, and pyloroduodenal duplications [3,7,8]. While our study focuses on three different and rare cases of enteric duplication cysts, we have encountered ileal and jejunal EDC amongst the cases, which can be considered relatively more common, yet due to their overlapping symptoms with other conditions, can be missed.

There is significant knowledge and awareness about enteric duplication cysts, yet they continue to pose a diagnostic dilemma and therapeutic challenge due to their rarity and variable clinical manifestations [2,4,7]. Generally, antenatally diagnosed neonates are evaluated postnatally irrespective of symptomatology, while those who are previously undiagnosed commonly present with signs of intestinal obstruction, feed intolerance, recurrent bilious/nonbilious vomiting, and nonspecific abdominal pain [8]. Gastrointestinal bleeding or perforation is usually associated with the presence of ectopic gastric or pancreatic mucosa within the EDC [9-11].

The variation in location of EDC with respect to the entirety of the gastrointestinal tract is the basis of the wide and sometimes complicated clinical signs, such as in our infant who, though having a suspicious antenatal diagnosis, due to lack of postnatal counselling and management, subsequently presented with complicated perforation peritonitis and a prolonged postoperative course, as well as our final case, who, though not having a prior diagnosis, presented with recurrent intussusception due to the EDC acting as a lead point. It can be corroborated that a varied location is a primary confounding factor in diagnosis. In the study by Erginal et al., documenting their single-center experience of 26 years, only 40 cases were identified [6]. Two-thirds of their patients were infants, with 21 out of 40 patients having EDC localized to the ileum, which, as per other literature, is the most common location, also highlighted in our neonate who had a classical ileal EDC.

Sheikh et al. have reiterated in their study that even investigative modalities may be misleading for diagnosis [7]. In their report, they encountered an eight-year-old who was diagnosed based on clinical and radiological investigations with complicated Meckel’s diverticulum, but clinical exploration revealed a complicated mid-ileal duplication cyst twisting on its own pedicle. This conundrum, though, was identified in the neonate who presented with nonspecific findings, which can be attributable to multiple conditions causing neonatal intestinal obstruction. Yet, with specific radiological imaging, it was surprisingly identified to be an EDC. 

Iijima documented a case where a neonatal EDC was initially mistaken for an ovarian cyst, illustrating how clinical and radiologic suspicion must be followed by postnatal correlation to ensure proper diagnosis [8]. Diagnosis, though suspected clinically, primarily relies on imaging modalities, from ultrasonography and plain radiographs, which can reveal signs of bowel obstruction or perforation, to more advanced imaging such as contrast-enhanced computed tomography and magnetic resonance imaging [9-12]. While upper and lower gastrointestinal contrast studies can be useful in the early evaluation to assess any communication between the duplication cyst and the bowel lumen, they are often difficult to interpret [11,12]. PET scans and nuclear imaging may occasionally be employed, particularly for detecting complications such as bleeding caused by ectopic gastric mucosa [13]. The confounding factor in the neonate in the present case series that was faced was due to the overlap of symptoms with other common conditions, such as malrotation with midgut volvulus and jejuno-ileal atresia. The strongly suggestive ultrasonographic finding of a cystic lesion changed the course of suspicion towards the rare possibility of an EDC, necessitating a higher investigation prior to surgical intervention. The infantile cases, though, required considerable rapid diagnostic approaches due to the presentation with peritonitis and intussusception, respectively. The availability of an antenatal report guided the process in the second case to consider CECT abdomen and pelvis, barring which it may have remained a diagnostic shock intraoperatively, causing difficulty in the decision-making process, while in the third case, the motive to look for a lead point due to recurrent intussusception aided in identifying the previously missed ileal EDC. This highlights the importance of the upcoming detailed antenatal diagnostic process, which can avoid the development of deadly complications.

In a study by Nebot et al., the author states that ultrasonography remains the investigation of choice for the diagnosis of an enteric duplication cyst with classically identifiable signs such as the “5-layer cyst wall” sign and the “Y” sonographic configuration of the muscle layer caused by the splitting of the shared muscularis propria between the cyst and the adjacent loop [14]. But advanced radiological imaging, such as CECT and MRI of the abdomen and pelvis, can be considered for anatomical delineation or in complex cases with doubtful diagnoses, though it is not essential in all patients [12-14]. Though ultrasound was confirmatory in all three of our patients, a CECT was done in the first two cases primarily to confirm the diagnosis and for better anatomical delineation for surgical intervention.

According to a case report by Kanna et al., prenatal diagnoses of duplication cysts are difficult, yet this plays a crucial role in improving neonatal outcomes and optimizing perinatal management [15]. With advances in ultrasound and fetal MRI, there has been an increase in the early detection of these anomalies, allowing for timely intervention to prevent complications such as obstruction, volvulus, perforation, or infection. In fact, prenatal imaging helps in differentiating EDCs from other cystic lesions, ensuring accurate diagnosis and appropriate postnatal management [8,13-15]. 

There is still significant debate about the timing of management in patients diagnosed antenatally. Dreznik et al., in their retrospective analysis of 16 children with enteric duplication cysts, noted that 11 were diagnosed antenatally, of whom 10 underwent elective surgery successfully at a median age of 1.3 years, with one patient requiring emergency surgery due to complicated EDC [16]. They recommend that asymptomatic, small duplication cysts in the small bowel of pediatric patients can be managed expectantly, and elective surgery can be done after the first year of age, but continuous and close follow-up is imperative due to the possibility of the development of complications [16]. Despite this, there is still disagreement on the timing of surgery, with our case series demonstrating all three children presenting with complicated EDC [1-3,6-9,16]. It is essential to understand that symptomatic EDC requires emergent management.

Surgical resection is the gold standard for management, whether an EDC is uncomplicated or complicated [8-11]. The choice of surgical approach depends on factors such as size, location, and presence of vascular involvement [14-16]. While the standard treatment of an enteric duplication cyst, complicated or not, involves complete surgical excision of the duplication cyst along with the contiguous segment of bowel, followed by end-to-end anastomosis, patient clinical stability and contamination intra-abdominally can alter the decision-making process, necessitating even placement of a stoma as a life-saving maneuver to decompress the bowel [2,3,6,12,13]. It is essential, though, that asymptomatic EDCs should also be managed electively as soon as possible so as to avoid untoward complications [7,18].

Holcomb et al., in a case series involving 96 patients with enteric duplication cysts, reported that these lesions often necessitate urgent surgical intervention due to complications such as mucosal ulceration, massive gastrointestinal bleeding, bowel perforation, and even, rarely, intussusception [9].

Blank et al. emphasize the surgical management of EDC, even in asymptomatic cases, to prevent potential short- and long-term complications, including malignant transformation, such as the development of adenocarcinoma, mucinous neoplasms, and, rarely, gastrointestinal stromal tumors (GIST) [13]. Small cystic or short tubular duplications are typically treated with segmental resection of the lesion along with the adjacent bowel [13,14]. In contrast, long tubular duplications are often managed by mucosal stripping via multiple incisions, as complete excision may result in short bowel syndrome [10,14-16]. In our study, however, all cases were successfully managed with complete surgical resection, which remains the simplest and most commonly expected management.

While the diagnosis and surgical management are evidence-based and follow a defined protocol, there has been significant debate on how an enteric duplication cyst should be diagnostically categorized, and this requires a definitive histopathological diagnosis in each patient who undergoes surgical intervention [1-3]. It is imperative that a record of the same is maintained, due to the rarity of the condition, the necessity for counselling, and for furthering scientific knowledge on any unusual findings identified with such a cyst [4-6]. According to Sharma et al., it is critical to maintain a diagnostic categorization to label an anatomical lesion as an enteric duplication cyst [1]. This evidence is supported by multiple studies as well [17-19]. The three primary characteristics, as per available literature, are (a) a well-developed smooth muscle coat; (b) mucosal lining found within some portion of the alimentary tract; and (c) contiguity to any segment of the alimentary tract [1-3,17-19]. These findings were noted intraoperatively and confirmed histopathologically in our cases, concluding the preliminary diagnosis.

The three operated cases not only demonstrate the complications associated with enteric duplication cysts but also highlight the necessity of an antenatal diagnosis, the importance of postnatal evaluation and management, and the imperative of definitive surgical management. This also brings physician notice to the varied clinical presentations with respect to age group and complications, highlighting the difficult process of maintaining diagnostic suspicion.

## Conclusions

It is essential to have clinical knowledge and scientific awareness about enteric duplication cysts, despite their general rarity, in order to procure an accurate diagnosis. This is particularly important, given its potential to present in diverse clinical forms across different age groups. If left untreated, it can lead to complications, occasionally catastrophic, such as perforation, obstruction, volvulus, uncontrolled hemorrhage, recurrent intussusception (as a lead point), and even subsequent malignant transformation, resulting in significant morbidity and mortality. While diagnostic and management protocols require constant update, postoperative vigilance is equally critical, particularly in complicated cases, to ensure good long-term outcomes. The limitation of this study was the small number of cases evaluated and managed. Further prospective studies with multiple cases and possibly even meta-analysis would aid in the development of better systematic protocols in the management of enteric duplication cysts.
